# The complex nature of research dissemination practices among public health faculty researchers

**DOI:** 10.5195/jmla.2019.524

**Published:** 2019-07-01

**Authors:** Rosie Hanneke, Jeanne M. Link

**Affiliations:** Assistant Professor and Information Services/Liaison Librarian, Library of the Health Sciences, MC 763, University of Illinois at Chicago, 1750 West Polk Street, Chicago, IL 60612, rhanneke@uic.edu; Assistant Professor and Head Librarian, Information Services and Research, Library of the Health Sciences, MC 763, University of Illinois at Chicago, 1750 West Polk Street, Chicago, IL 60612, jlink@uic.edu

## Abstract

**Objective:**

This study explores the variety of information formats used and audiences targeted by public health faculty in the process of disseminating research.

**Methods:**

The authors conducted semi-structured interviews with twelve faculty members in the School of Public Health at the University of Illinois at Chicago, asking them about their research practices, habits, and preferences.

**Results:**

Faculty scholars disseminate their research findings in a variety of formats intended for multiple audiences, including not only their peers in academia, but also public health practitioners, policymakers, government and other agencies, and community partners.

**Conclusion:**

Librarians who serve public health faculty should bear in mind the diversity of faculty’s information needs when designing and improving library services and resources, particularly those related to research dissemination and knowledge translation. Promising areas for growth in health sciences libraries include supporting data visualization, measuring the impact of non-scholarly publications, and promoting institutional repositories for dissemination of research.

## INTRODUCTION

The dissemination practices of public health researchers are complex and wide-ranging. A national survey of public health researchers found that one third usually or always disseminated their findings to non-research audiences in a summary format, such as an issue brief [1]. However, the most common mode of dissemination is through scholarly journals and academic conferences, and dissemination outside of traditional academic routes is done less frequently and often on an ad hoc basis [2]. Barriers to disseminating public health research to local and state health departments include organizational factors (lack of time, money, and academic incentive) as well as individual factors (uncertainty as to how best to disseminate findings or which organizations to target) [3].

Research into the information practices and needs of users in public health to date has predominantly focused on practitioners working outside of academia. Multiple authors have found that public health workers experience multiple barriers to accessing necessary information. This population relies on a diverse collection of information formats, including heavy use of grey and unpublished literature [4–8]. There have been few investigations of the information behavior and needs of academic public health researchers (i.e., university-affiliated faculty) in the library literature. Librarians at the University of Minnesota observed that the “breadth of topics and scale of work” involved in public health research by faculty “require a diverse range of information types” [9]. The information needs of faculty researchers in public health cannot be assumed to be the same as those of public health practitioners, due to the variations in workflow, priorities, and resources among these groups [9–11]. Therefore, it is important to investigate the needs and behaviors of faculty more thoroughly.

Ithaka S+R’s ongoing Research Support Services (RSS) program uses in-depth interviews with faculty researchers to investigate their research practices and preferences [12]. This process aims to understand faculty information needs better as well as how these needs vary by discipline [13]. Previous RSS studies have explored the research needs of scholars in history, agriculture, chemistry, art history, and religious studies.

Between 2016 and 2017, Ithaka S+R led a team of librarians from 7 institutions who conducted interviews with faculty members who did research in public health. The final report created by Ithaka S+R, including links to local institutional reports, is available online [13]. The University of Illinois at Chicago (UIC) Library participated in this study. As of spring 2015, there were 316 faculty appointments at the School of Public Health (SPH), including 75 primary (100%) appointments, totaling 86.44 full-time equivalent faculty members. A local report including a broad summary of findings from the UIC SPH is presented elsewhere [14]. In addition to their local report, the research team at the University of Minnesota, which also participated, has published an article that summarizes their findings [9].

The Ithaka S+R RSS study is the first to use semi-structured interviews to comprehensively explore the information practices of public health faculty researchers. This paper will contribute to addressing this gap by placing the authors’ findings from interviews with UIC SPH faculty in the context of the ongoing scholarly conversation. It provides a close examination of the varied types of information produced and audiences targeted by public health faculty at UIC in the process of disseminating their research in order to identify areas for growth and improvement in library services. Some portions of this paper have been adapted from UIC’s local institutional report.

## METHODS

UIC’s Institutional Review Board approved the authors’ claim for exemption from review. We conducted semi-structured interviews with twelve faculty members from the SPH. Seeking to recruit participants from all ranks and all four academic divisions of the SPH (Community Health Sciences [CHS], Environmental and Occupational Health [EOHS], Epidemiology and Biostatistics [EPI-BIO], and Health Policy and Administration [HPA]), we gathered faculty names from the SPH website and sent each individual an email requesting their participation. Faculty members from all four academic divisions were willing to speak with us. We also used convenience and snowball sampling to recruit additional participants beyond the respondents to our initial emails. Categorization of interview participants by rank and division is provided in Appendix A.

Participants agreed to meet individually, in person, for an interview of up to sixty minutes. Using a semi-structured interview guide developed by Ithaka S+R (supplemental Appendix B), each interview was conducted by one of the two authors. None of the interview questions directly asked participants about the library. Instead, the questions probed for broader information about research habits and preferences to explore potential opportunities for growth rather than biasing conversation toward current library services. All interviews took place between January 23 and March 7, 2017. We recorded the interviews using an audio recorder, and the interviews were transcribed by a third party. We deleted the audio files once the transcripts had been checked for accuracy and anonymized.

We coded the transcripts in three stages and used Dedoose software to mark up excerpts and tag them with applicable codes. Choosing three transcripts at random, both authors coded the same three transcripts independently. We compared our coded transcripts, and our comparison gave rise to an initial list of core themes and codes. Using this list, we coded a fourth transcript together aloud to discover and resolve any differences in how we interpreted and applied the codes. The twelve transcripts were then divided between the two authors to be coded individually. Core themes that emerged from this process were:

- “Information Discovery”: the processes of searching for and accessing secondary information to support the participant’s own research;
- “Research Dissemination”: the processes of communicating research findings to various audiences, including but not limited to public health academics, practitioners, and community partners;
- “Grey Literature”: discussion of nontraditional information formats, including data sets, research reports, government documents, and others;
- “Interdisciplinary Collaboration”: public health faculty working with scholars or professionals from different fields of expertise toward a common goal; and
- “Research Data Management”: plans or rules of governance for organizing information for the sake of publication or preservation and ease of access or sharing.

Two themes, Research Dissemination and Grey Literature, are the focus of this paper.

## RESULTS

Public health faculty researchers at UIC described producing a wide variety of publication types in the process of disseminating their research. Their findings were disseminated across both scholarly and non-scholarly sources; a major influencing factor was that many faculty hoped to reach and impact multiple audiences. These included a traditional academic audience and extended to public health practitioners, government agencies, policymakers, and community partners. Some of these groups collaborated with faculty on research projects and thus partnered with them throughout the research process. The differences among these audiences translated to a variety of formats in which research data were described, visualized, and distributed.

### Within academia

As might be expected, the most common outlet for faculty research was traditional scholarly peer-reviewed journals. Participants overall indicated that this was the standard expectation in their field. Some faculty members working in specific subdisciplines, such as industrial hygiene, said that there were just a few journals in which they would consider publishing their work. Others, whose work was self-described as more interdisciplinary than strictly falling within the borders of public health, published in a more diverse collection of journals. One participant noted that “it’s quite variable…which kind of journal it’s going to be depending on the topic. The topics I publish on vary quite a bit.”

Faculty members published in journals across the health sciences, some more clinically focused than public health journals, as well as in social sciences journals. Individuals who attempted to publish outside of traditional public health journals experienced some challenges. One participant described “starting to try to publish in the infection control literature, which are more clinically oriented” than other public health journals. She continued, “I’ve struggled to get my articles into those…because I don’t normally write for that audience. I haven’t figured out their jargon yet.” A faculty member in health policy expressed a desire to improve the visibility and discoverability of her studies that were published in public policy journals, since these journals are not indexed in PubMed or other health sciences databases typically searched by public health scholars.

Faculty members who conducted research using qualitative methods indicated that it was often difficult to publish this type of research in scholarly journals. One individual noted that “there aren’t a lot of journals in which you can publish this…It isn’t easy research to publish, really, because it’s so qualitative.” This participant elaborated on what he believed to be a “larger issue within the sciences” that led to these challenges, noting that “in a lot of ways people still question the validity of qualitative work.” He added that “fortunately enough…because of those challenges, now there are very specific CBPR [community-based participatory research] kind of related journals” that are receptive to qualitative research, so he and other qualitative researchers could focus their efforts on publishing in those outlets.

The expectations for promotion and tenure did not factor significantly into our discussions about publication, as all but one of our interview participants were either on a clinical (nontenure) track or had already achieved tenure. The single tenure-track/pre-tenure faculty member who spoke with us acknowledged that this was a major influence on choosing a venue in which to publish:

You have to be very strategic about where you publish, what you do…[I try] my best to get into really good, reputable journal outlets…what I try to do is a good balance of really high impact but also journals where I know it could…have better influence on individuals.

A tenured professor with experience as a promotion and tenure committee member mentioned that the SPH had recently begun to “expand its definition” of what qualified as a scholarly publication, explaining that:

Peer review is important, whatever it is. But—blogs, and webinars, and websites, I include all those sorts of things on my CV now. I think it’s perfectly reasonable to include them in promotion packages. It shows reach. It shows impact and outcomes, especially when you’re doing community-engaged kind of research.

Another participant said that having full tenure allowed her more freedom to publish outside of traditional academic journals. She noted that scholarly journals were “not my primary outlet anymore…Of course, I sort of am fortunate in that I already have my full tenure and I’m a full professor. I don’t really need to worry about that publishing record.”

### Beyond academia: practitioners, policymakers, and community partners

Many participants described attempts to disseminate their research or scholarship directly to practitioners in addition to academic channels. The degree of effort put into dissemination of findings beyond scholarly journals was intensive among those interviewed. One faculty member balked at calling himself a researcher, instead describing his work as a more pragmatically oriented “scholarly inquiry” because it was “more aligned with practice if you look on that continuum than research, which is more abstract…It’s very much related to what do people use and need in practice.”

Others did not mention the concept of scholarly inquiry but nonetheless had a strong orientation toward producing research that had an immediate impact on practice. For example, one individual reflected, “I’m a pretty applied person. I’m hoping it doesn’t just stay in the academic literature.” Another noted, “I always want to bring in what’s happening in practice that’s complex and unique into then, how can academia support it? Really for my entire career, I’ve always danced between the two worlds.” For this reason, some individuals said that they sought feedback on their manuscripts from practitioners in lieu of—or in addition to—undergoing the traditional scholarly peer-review process.

When partnering with local organizations and health departments, researchers frequently translated findings into training materials for these organizations, including webinars, flyers, and websites. One participant indicated that her research team’s findings were typically disseminated via partner organizations’ websites rather than websites hosted by UIC or scholarly journals. In some cases, collaborators were invited to join as coauthors. One participant noted, “I always extend the opportunity for individuals of those agencies and programs to also join as co-authors. I think that’s really important to make sure that they feel as much invested in the process as I did.”

Creating a research product that can be applied directly to public health practice, especially when created in addition to a scholarly journal article, can be a time-consuming process. Still, multiple participants mentioned the importance of ensuring that their research had an impact on practice. One faculty member reflected that her staff enjoyed creating “practitioner-oriented products. They love doing that because they’re talking with their field that way as opposed to just talking to academics.”

Another faculty member described a moral motivation behind creating products that were immediately useful to public health practitioners, saying, “it’s the right thing to do, in my opinion, to take action, but it’s laborious and it’s more work…to make sure there’s some kind of resolution and action from what you’re doing.” She expressed a strong desire to “spend more time making meaning out of” her research findings, adding that her goal was “not just to collect it for the information’s sake.”

Many public health faculty researchers aimed to influence policy and legislation. To this end, they disseminated their findings in multiple formats. These included not only traditional scholarly journal articles, but also extended to reports, policy briefs, and web documents, with the intention of reaching a wider audience of decision-makers and health policy influencers. One participant described the process in the context of writing for multiple audiences, which she noted can be challenging:

We have so many kinds of writing that we do in public health. I will go next week to the Hill and meet with Congresspeople and I need something in my hand that resonates with them, that is framed from their perspective. That’s different from the thing I write for whatever journal. It’s different from what we’re going to put on our website. Every single one of these things has different kinds of thinking about it and writing about it. I don’t have a place to go that helps me with all of that.

Another faculty member said that “A lot of what we do is to bring the science to the policy debate,” for example, writing environmental health reports “for various advocacy groups that will post it on their webpages or whatever. They’re not peer reviewed. Hopefully they are accurate and balanced, so that in that way, scholarly, but they’re not the scholarly literature.”

One major impetus for publishing research in multiple formats was the desire to meet decision makers where they are. For maximum impact, one participant explained, research must be disseminated outside of academic journals.

We’re increasingly finding that all of our academic papers, it doesn’t matter what we put out there. Unless we translate them into manageable products, like short briefs, fact sheets, whatever, they’re not going to get used by decision makers. Even if we are doing a manuscript, I always want a companion product to go along with it.

Further, this information could be published more rapidly than in the academic publishing cycle:

It’s becoming increasingly typical in the public health policy field, for sure. Anyone who’s doing work with the foundations, the foundations in particular are much more keen on rapid dissemination. Again, getting information out in a rapid way to inform decision making and not wanting to wait for the academic publishing cycle.

Several faculty researchers described their involvement in community-based participatory research, an approach that focuses on collaboration and equal partnership with community members rather than a more traditional investigator-participant relationship [15]. These faculty members expressed a desire to incorporate their research findings into products that could be presented to community partners as a resource for their own use. One participant described this perspective as “a little anti-academic,” saying that:

If I’m spending time in the community, and they’re spending time with me, me getting a publication out of this is not my goal…I have never actually been in a position where I simply wrote an article, and there wasn’t anything back to the community. It is just not in my nature to do that.

UIC SPH faculty published myriad products for community partners from their research data, including flyers, infographics, presentations, and educational comic books. A traditional academic paper was often prepared alongside or following the preparation of these community resources.

One participant described the multiple formats in which her research team provided community partners with information in the past, including “short one-page summary reports. More often than that, we will use, now the more infographic kind of one-pager.” She explained, “it’s not just dot-point information, there’s figures on there, and little graphics, and things like that, that people seem to feel more comfortable with.”

Faculty noted that community partners were typically invited to review these products and provide feedback. One participant noted that “the target population [for a publication] is often the people who are participating in the process. It’s a summary of their results, and they review it, and tell me whether…it’s relevant or not.” The process was described as follows:

We’ll ask them, “Is this meaningful to you? How else would be a better way?” We feel that we collaborate on our instruments, we get the information, we process it, and then we will give back the information in the format that the community tells us to. “We would like to see this more in pictures, or we would like to understand this…Can you make a chart for us?”

Another said that such feedback helped not only to ensure that the information presented was correct, but also to strengthen any scholarly article written from the findings. She said that this feedback helped “develop the recommendations that I would also write within an article, and through that process, I think it makes a much more solid piece.”

The faculty members with whom we spoke were largely unfamiliar with UIC’s institutional repository, INDIGO, as well as the general concept of disseminating their publications or data via a repository. One participant said that the issue simply “hasn’t come up” and asked, “What’s an example of a repository?” When asked why she had not before made her data available in a repository, another participant replied, “I have no idea. It’s never come up.” Another expressed doubt as to how he could make his data publicly available when they could not be anonymized. Conversely, two of the twelve faculty members were familiar with the process but described various challenges in using repositories. These included a dislike for UIC’s repository platform and frustration with a long response time from the international Inter-university Consortium for Political and Social Research repository.

## DISCUSSION

Research dissemination in public health is a complex process due to the desire of many researchers to impact policy and practice directly. When speaking about research dissemination, one recurrent theme among the scholars we interviewed was their intense passion for public service. Several faculty members expressed a deep-seated desire to effect positive change in the community, the nation, and beyond. This desire has a direct influence on their dissemination practices, as they wanted their findings to have broad impact and to reach far beyond their peers in academia.

Because of tenure obligations, this motivation often puts an added pressure on tenure-track faculty researchers who not only must publish articles in academic journals, but also wish to produce additional outputs for other audiences, effectively doubling their workloads. It has been found through analyzing university promotion and tenure documents that faculty are not incentivized to produce “nontraditional” research outputs like blog posts, policy briefs, and newsletters, nor are there established metrics for evaluating scholarly contributions to the public good [16].

One major role for the librarian in the research cycle that was supported by our findings was that of measuring impact. In addition to tenure-track faculty who rely on it for promotion documentation, researchers used impact information to justify requests for external funding as well as to demonstrate the successes of their previous efforts. Measuring the impact of research disseminated outside of traditional scholarly journals is an altogether different task from the straightforward methods used for scholarly journal articles, such as citation counts and h-indexes, and often is challenging [17].

Altmetrics, once defined strictly as online impact of a publication as demonstrated on social media, now includes any measure of impact beyond traditional metrics and is a promising way for librarians to provide support in this area [18]. For example, measuring the download counts for a research report is a simple yet effective means of conveying the publication’s reach. If a publication receives attention through tweets or other social media mentions, this information is also worth noting.

The development of metrics for determining the impact of grey literature publications, such as the “modified citation analysis” method proposed by Sibbald and coauthors, provides even more ways to measure impact and merits further examination [19]. Another method, the web impact report (WIRe), proposes measuring the impact of grey literature by website mentions, content analysis (how and why the information was used), and a ranking of the value of the citing websites [20].

These methods nonetheless still focus on measuring online impact. Real-world impact of public health information products, such as whether a pamphlet promoting hand hygiene or monthly breast self-examinations actually improves those practices in the community, can still seem an elusive metric. The Becker Medical Library Model for Assessment of Research Impact provides an extensive list of quantifiable “impact indicators,” including measures of community benefit and changes to legislation and policy [21]. An example from humanities and social sciences, the HuMetricsHSS initiative, encourages the use of metrics to measure a scholar’s “progress toward embodying five values that…are central to” those disciplines, including equity (“the willingness to undertake study with social justice, equitable access to research, and the public good in mind”) and community (“the value of being engaged in one’s community of practice and with the public at large and also in practicing principled leadership”) [22]. Such value-based metrics echo the public service orientation of many of our study participants.

These indicators can be used to demonstrate a comprehensive picture of research impact when presented alongside citation counts and other traditional impact measures. Such frameworks are not without limitations and, like traditional metrics, often do not convey the full picture of a publication’s reach. Nonetheless, they provide the quantitative measures that faculty need and offer a promising step toward a wider-lens view of how research impact can be observed and demonstrated. This also presents the opportunity for librarians to initiate a general conversation with faculty about how impact is measured, as well as the limitations of both traditional and more recently developed metrics. Gutzman and coauthors recently presented a description of research evaluation service models at seven health sciences libraries that further expands on the potential opportunities for growth in this area [23].

Studies of knowledge translation have explored the barriers and facilitators to putting research knowledge into action [24]. It has been found that while public health researchers value dissemination to non-research audiences, their findings are still primarily disseminated through traditional academic routes, namely, scholarly journals and conferences [25]. Knowledge translation interventions are most effective when tailored and targeted to a specific end user [26]. However, many public health researchers experience significant challenges in this process. Researchers at large federal agencies such as the Centers for Disease Control and Prevention or National Institutes of Health often have access to health communication or public relations professionals at their organizations to help them create information products targeted at specific audiences, for example, policymakers or community partners [1]. Researchers at universities are far less likely to have access to such resources. Several faculty participants mentioned the need for someone to help develop materials such as infographics and fact sheets.

Helping to close gaps in the process of translating research into action is an area of further exploration for librarians. Tabak and coauthors explain that “researchers may not be ideal disseminators of their work” and that they would benefit from a third party to “bridge this gap and facilitate the handoff of information” [27]. Along with communications professionals, the library might help fill this role. If librarians themselves do not feel that they have the expertise to teach dissemination tools and techniques to faculty, they can position the library as a central campus location for these conversations, for example, by hosting workshops led by the library’s communications professionals or by graduate students, faculty, or staff from other units who specialize in communication, marketing, or public relations. These individuals can provide information related to disseminating research in multiple venues, from community-targeted publications to mass media.

The library can thus be used as a central locus for reaching faculty not just in public health, but across campus. Librarians at the New York University (NYU) Health Sciences Library have led such an initiative by hosting a data visualization critique clinic and data-related classes, thereby acting as a “data hub” to bring together researchers from across campus and create a forum in which constructive feedback can be shared and a sense of community is fostered among researchers [28, 29].

Institutional repositories are ideal platforms for faculty to share research that is not published in traditional scholarly venues, due to permanent, open-access hosting with persistent uniform resource locators (URLs). However, the majority of our interview participants were not familiar with UIC’s repository, INDIGO. This resembled the findings of researchers at the University of Florida, who learned that agriculture faculty infrequently used their repository and were often not aware of the full potential of this resource [30]. Faculty might focus on publishing in venues where they know they can reach a specific audience and might not be concerned with finding a central, permanent hosting site for their research outputs.

Nevertheless, institutional repositories “could make a substantial difference in ensuring grey literature’s preservation, increasing its reach, and, in many cases, providing a form of legitimacy to these items published outside traditional realms” [31]. A study at one institution found that grey literature in its repository was downloaded at a significantly higher rate than were articles, books, or book chapters [32]. It is up to the library to spread the word about this function of the institutional repository and the importance of permanent hosting for all research outputs, including data sets and other types of grey literature. Many users may not realize that institutional repositories are indexed by Google Scholar and, therefore, improve the broad discoverability of their research. The library can also take this opportunity to educate faculty about sharing their work legally and in accordance with publisher agreements [9].

Because time can be a significant barrier to faculty motivation for self-archiving in repositories, the library should help streamline the process as much as possible. Troll-Covey suggests actively seeking out grey literature produced on campus, as well as coordinating submission with faculty’s annual report process, in which they must report their publications to the university [33]. These and other aspects of nontraditional research dissemination should factor into scholarly communication conversations, which sometimes are limited to the publication and impact of academic journals and monographs.

Finally, a natural consequence of the fact that public health research is published in nontraditional outlets, such as research reports and issue briefs, is that public health students must learn the skills that are necessary to locate these publications. If they are not already well versed, public health librarians should educate themselves about these information sources, because researchers in public health “may need more in-depth training on grey literature search strategies than other professions” [9]. Librarians must devote energy to designing pathways, instruction sessions, and other resources to help users locate the information themselves.

Librarians who lack confidence in their own knowledge of locating grey literature may find it useful to seek continuing education opportunities through several professional organizations’ offerings. Organizations that have recently promoted such opportunities include the Medical Library Association and its regional chapters [34, 35], the National Network of Libraries of Medicine [36], the American Public Health Association [37], and government entities such as the Centers for Disease Control and Prevention (CDC TRAIN) [38, 39] and the Substance Abuse and Mental Health Services Administration [40]. Those librarians who are knowledgeable about locating grey literature are encouraged to share their knowledge with fellow librarians as well as with public health academics and professionals by offering continuing education courses and webinars or presenting papers and posters at professional meetings.

By developing the required knowledge for grey literature instruction in public health, librarians will support the curriculum as well as the future information literacy needs of public health students, whether they ultimately choose to work in academia or practice. While librarians should know about and recommend helpful grey literature resources (e.g., DisasterLit, PHPartners, OpenGrey), multiple authors have emphasized the need to teach general information literacy skills that help users locate and evaluate these publications rather than teaching only how to search specific resources [41, 42].

### Limitations and methodological reflections

None of the interview questions directly asked participants about the library. Some participants might have mentioned the library—or omitted negative reflections with respect to the library—due to preexisting relationships with one or both authors or simply because they knew the interviewer was a librarian. At the same time, the omission of questions about the library might have allowed a broader view of participants’ behaviors and preferences, unbiased by their conceptions of the boundaries of what constitutes library services.

We attempted to recruit faculty from all ranks; however, only one of our interview participants was an assistant professor on the tenure track. All other participants either had been awarded tenure or were in nontenure-track positions. It is possible that this influenced our findings concerning research dissemination practices. While the faculty interviewed here described a preference for disseminating their research in myriad formats other than the scholarly journal article, pre-tenure faculty may in fact favor this more traditional format due to the requirements imposed by promotion and tenure norms. It should be noted, however, that the single tenure-track faculty member we interviewed was enthusiastic about publishing in multiple formats for wide audiences, not exclusively in scholarly journals.

It was not possible to interview faculty researchers from all the varied subdisciplines in public health. Our analysis reflected attitudes and practices at one institution. Our analysis was not representative of all public health researchers but was an exploration of those subdisciplines represented by the interview participants at our institution.

### Implications for library services

The information gleaned from these interviews provided the authors with several actionable ideas that could be implemented at their library or at other institutions to better reach faculty in public health as well as those in other health sciences disciplines with similar needs. These ideas, supported by previous research, include:

- explore ways to serve as a campus hub to support data visualization and other techniques that are useful in communicating research findings to community partners, decision makers, and other nonacademic audiences and seek outside experts if librarians do not have expertise;
- investigate and advertise support for nontraditional research impact measures;
- promote the use of institutional repositories for research dissemination; and
- facilitate the discovery of grey literature, pursue educational opportunities in grey literature searching where they exist, or help to develop and deliver such education for other librarians.

The diverse information formats employed by public health faculty in the process of disseminating their research challenge us to think innovatively when developing and improving support services for these scholars. Librarians must educate themselves about the traditional scholarly information sources of the discipline, but these make up only a small portion of faculty members’ needs. These findings have wide implications for librarians, underlining a critical need for education, professional development, and research on all aspects of both scholarly and grey literature in public health, from production and dissemination to organization and discoverability.

## SUPPLEMENTAL FILES

Appendix A Division and rank of interview participants

Appendix B Semi-structured interview guide
