# Supplementary material for: The complex nature of research dissemination practices among public health faculty researchers
Source: J Med Libr Assoc. 2019 Jul 1;107(3):341–51. doi: 10.5195/jmla.2019.524 (PMC6579591; doi:10.5195/jmla.2019.524)
Supplement: Appendix A [file jmla-107-341-s001.pdf]

JMLA Journal of the Medical Library Association

SC
Supplemental content to
J Med Libr Assoc. Jul;107(3):dx.doi.org/10.5195/jmla.2019.524
www.jmla.mlanet.org
© Hanneke, Link 2019

The complex nature of research dissemination practices among public health faculty researchers

Rosie Hanneke, MLS, AHIP; Jeanne M. Link, MLS, MS

APPENDIX A

Division and rank of interview participants

	Community Health Sciences	Environmental and Occupational Health Sciences	Epidemiology and Biostatistics	Health Policy and Administration
Assistant professor (tenure-track)	1			
Associate professor (tenured)	1	1		
Professor (tenured)		2	2	1
Research assistant professor (nontenure-track)		1		
Clinical assistant professor (nontenure-track)	2			
Clinical associate professor (nontenure-track)				1
