# Supplementary material for: The complex nature of research dissemination practices among public health faculty researchers
Source: J Med Libr Assoc. 2019 Jul 1;107(3):341–51. doi: 10.5195/jmla.2019.524 (PMC6579591; doi:10.5195/jmla.2019.524)
Supplement: Appendix B [file jmla-107-341-s002.pdf]

JMLA Journal of the Medical Library Association

SC
Supplemental content to
J Med Libr Assoc. Jul;107(3):dx.doi.org/10.5195/jmla.2019.524
www.jmla.mlanet.org
© Hanneke, Link 2019

The complex nature of research dissemination practices among public health faculty researchers

Rosie Hanneke, MLS, AHIP; Jeanne M. Link, MLS, MS

APPENDIX B

Semi-structured interview guide

Research focus

1. Describe your current research focus/projects:
    a. How is your research situated within the field of public health?
    [Probe for which subdiscipline(s) their work aligns with and whether they engage in interdisciplinary work within public health and/or with other fields]
    b. What led you to this? (What is your background?)

Research methods

2. What research methods do you currently use to conduct your research?
    [Probe for whether these methods are typical for public health scholars]
    a. Is this methodology common for this line of research?
    b. Have you conducted systematic reviews, rapid reviews, or other types of literature reviews as part of your research?
    c. Do you collaborate with others as part of your research?
    [If yes, probe for what these collaborations entail, who typically works on them, what the division of work is, and how information pertaining to the project's research is created and stored]
    d. What does this collaboration look like? What are the logistics?
    e. Do you employ research assistants? If so, can you talk about how delegation works?

3. Does this project involve collecting data?
    What is it you are trying to learn?
    What are you trying to reveal/discover?
    a. What kinds of data does your research typically elicit?
    b. How do you incorporate these data into the papers or presentations that come out of your research?
    [Probe for whether they use data visualization tools] Examples?
    c. How do you manage and store these data for your ongoing use?
    Is anyone in particular in charge of this?
    If you have a specific protocol or system for naming, organizing, and storing data, is it one you adopted or developed for yourself?

Supplemental content to
J Med Libr Assoc. Jul;107(3):dx.doi.org/10.5195/jmla.2019.524
www.jmla.mlanet.org
© Hanneke, Link 2019

4. Beyond the data your research produces, what kinds of information do you rely on to do your research?
   Are you building upon previously presented research—either yours or someone else's?
   a. How do you locate this information?
      [If not explicitly stated, probe for where they locate the information]
   b. How do you manage and store this information for your ongoing use?
   c. Do you experience any challenges working with this kind of information?

5. Can you tell me about a past or ongoing research project where you faced challenges in the process of conducting the research?
   a. Describe these challenges. What does that look like?
   b. What could have been done to mitigate these challenges?
   c. Are there any other challenges you regularly experience when conducting your research?

6. How do you keep up with trends in your field more broadly?
   a. How do you become aware of recently published research?
   b. How do ideas get shared across collaborating researchers?
   c. Where do creative discussions happen that help you develop ideas?

Dissemination practices

7. Where do you typically publish your research in terms of the kinds of publications and disciplines?
   a. Do you disseminate your research beyond scholarly publications?
      [If so, probe for where they publish and why they publish in these venues]
      Do expectations for promotion and tenure influence these decisions?
      (i.e., to publish, where to publish, or to disseminate research other than in publications, such as presenting papers or posters at conferences)
   b. How do your publishing practices relate to those you consider typical to your discipline?

8. Have you ever deposited your data or final research products in a repository?
   a. If so, which repositories and what have been your motivations?
      (i.e., required, for sharing, investment in open access principles)
   b. If no, why not?

   Does your research have more of an application to the work of academics, practitioners, or both? (Or another group?)

Future and state of the field

9. What future challenges and opportunities do you see for [your research area and] the broader field of public health?
   Potential interdisciplinary collaboration?

JMLA Journal of the Medical Library Association

Supplemental content to
J Med Libr Assoc. Jul;107(3):dx.doi.org/10.5195/jmla.2019.524
www.jmla.mlanet.org
© Hanneke, Link 2019

10. If I gave you a magic wand that could help you with your research and publication process, what would you ask it to do?

Follow-up

11. Is there anything else about your experiences as a scholar of public health and/or the public health discipline that you think it is important for me to know that was not covered in the previous questions?
